# Malaria impact of large dams at different eco-epidemiological settings in Ethiopia

**DOI:** 10.1186/s41182-017-0044-y

**Published:** 2017-02-24

**Authors:** Solomon Kibret, G. Glenn Wilson, Darren Ryder, Habte Tekie, Beyene Petros

**Affiliations:** 1 Ecosystem Management, School of Environmental and Rural Science, University of New England, Armidale, NSW 2351 Australia; 2 Department of Zoological Sciences, Addis Ababa University, PO Box 1176, Addis Ababa, Ethiopia; 3 Department of Microbial, Cellular and Molecular Biology, Addis Ababa University, PO Box 1176, Addis Ababa, Ethiopia; 4 Present address: Program in Public Health, University of California, Irvine, CA 92697 USA

**Keywords:** Malaria, Mosquito breeding, *An. arabiensis*, *An. pharoensis*, *An. funestus*, Water management, Dams, Irrigation, Africa

## Abstract

**Background:**

Dams are important to ensure food security and promote economic development in sub-Saharan Africa. However, a poor understanding of the negative public health consequences from issues such as malaria could affect their intended advantages. This study aims to compare the malaria situation across elevation and proximity to dams. Such information may contribute to better understand how dams affect malaria in different eco-epidemiological settings.

**Methods:**

Larval and adult mosquitoes were collected from dam and non-dam villages around the Kesem (lowland), Koka (midland), and Koga (highland) dams in Ethiopia between October 2013 and July 2014. Determination of blood meal sources and detection of *Plasmodium falciparum* sporozoites was done using enzyme-linked immunosorbent assay (ELISA). Five years of monthly malaria case data (2010–2014) were also collected from health centers in the study villages.

**Results:**

Mean monthly malaria incidence was two- and ten-fold higher in the lowland dam village than in midland and highland dam villages, respectively. The total surface area of anopheline breeding habitats and the mean larval density was significantly higher in the lowland dam village compared with the midland and highland dam villages. Similarly, the mean monthly malaria incidence and anopheline larval density was generally higher in the dam villages than in the non-dam villages in all the three dam settings. *Anopheles arabiensis*, *Anopheles pharoensis*, and *Anopheles funestus s.l.* were the most common species, largely collected from lowland and midland dam villages. Larvae of these species were mainly found in reservoir shoreline puddles and irrigation canals. The mean adult anopheline density was significantly higher in the lowland dam village than in the midland and highland dam villages. The annual entomological inoculation rate (EIR) of *An. arabiensis*, *An. funestus s.l.*, and *An. pharoensis* in the lowland dam village was 129.8, 47.8, and 33.3 infective bites per person per annum, respectively. The annual EIR of *An. arabiensis* and *An. pharoensis* was 6.3 and 3.2 times higher in the lowland dam village than in the midland dam village.

**Conclusions:**

This study found that the presence of dams intensifies malaria transmission in lowland and midland ecological settings. Dam and irrigation management practices that could reduce vector abundance and malaria transmission need to be developed for these regions.

## Background

The sub-Saharan Africa region is ranked lowest in the world for average water withdrawal [1], suggesting the pressing need for targeted development of water resource infrastructure. New water storages are currently being extensively developed to help improve the region’s food security and promote sustainable economic development [2, 3]. However, the link between dams and malaria has been widely recognized as a public health challenge [4–6] which could hamper the intended advantages provided by these water infrastructures.

Ninety percent of the global malaria burden occurs in sub-Saharan Africa, resulting in transmission and disease management being a leading public health challenge [7]. With the current high level of dam construction in the region [8], links between the spatial distribution of dams in the landscape and malaria outcomes must be better understood for an assessment of any potential negative public health outcomes from dam development. Previous studies have indicated that dams increase malaria by providing breeding sites for malaria-transmitting mosquitoes in areas with unstable/seasonal malaria [9–19]. For example, a study around the Akosombo Dam in Ghana documented a 20% increase in malaria incidence in populations within a 3-km radius of the reservoir compared with those residing more than 7 km from the reservoir [17]. The occurrence and persistence of shallow shoreline puddles around the edge of the reservoir providing breeding habitats for the primary malaria vector species, *Anopheles gambiae*, were indicated to underpin the increased malaria incidence [20].

A number of environmental factors determine the degree of intensity of malaria transmission in Africa. Elevation has long been known for its effect on malaria transmission, mainly due to its influence on ecological and climatic drivers. A study in Tanzania found that malaria prevalence decreases by 21% in every 100 m increase in elevation [21]. Higher temperatures and other ecological characteristics associated with lower altitudes have been indicated to support higher rates of malaria transmission in the lowlands than in the highlands, which are considered as epidemic-prone.

Although dams can increase malaria in unstable areas (i.e., areas with seasonal malaria), it is not clear whether the impact of dams on malaria varies in different ecological settings. As Africa is experiencing a new era of dam building, with numerous dams planned or currently under construction [5], understanding the link between dams and malaria transmission across different eco-epidemiological settings is crucial in order to devise malaria control strategies and enable appropriate allocation of limited resources for intervention around water resources development schemes.

The present study assessed the link between three dams and malaria at different eco-epidemiological settings in Ethiopia. The objective of this study was to identify mosquito breeding sites and compare adult and larval abundances around three dams in highland, midland, and lowland settings of Ethiopia. This study aims to compare the malaria situation across elevations and proximity to dams. Such information may contribute to better understand how dams affect malaria in different eco-epidemiological settings.

## Methods

### Study area

**Fig. 1:**
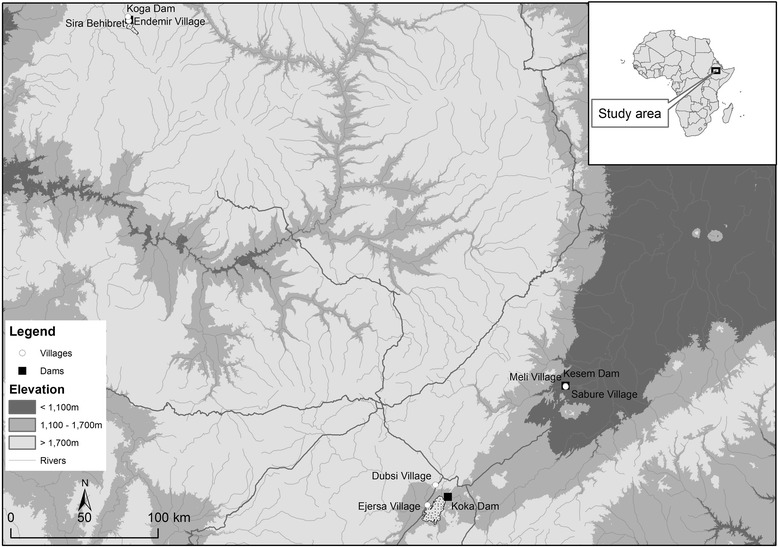
The study area in Ethiopia, showing the lowland Kesem Dam (912 m above sea level in the Rift Valley), the midland Koka Dam (the middle course of the Ethiopian Rift Valley, 1551 m asl), the highland Koga Dam (1950 m asl), and the dam and non-dam villages near each of these sites

This study was carried out around three dams in Ethiopia (Fig. 1): Kessem Dam (912 m above sea level (asl)), Koka Dam (1551 m asl), and Koga Dam (1,950 m asl). One village located within 5 km from the reservoir shorelines and another located farther away (>5 km) but not in downstream direction were selected for this study at each dam site. Generally, villages >5 km downstream of the dam were excluded as they are affected by water releases from the dam.

Kesem Dam (9°13′60′′ N, 40°6′0′′ E), hereafter referred to as the lowland dam, is located on the Awash River in the Ethiopian Rift Valley in east-central Ethiopia, 220 km from Addis Ababa. Its capacity is 500 million m^3^, and the primary use of its water is for irrigation of sugarcane crops that cover 20,000 hectares of floodplain downstream of the dam. The region is classified as arid with long-term average rainfall between 500 and 600 mm per year and a mean annual temperature of 26 °C (National Meteorological Agency, unpublished report). Sabure (932 m asl), hereafter referred as the lowland dam village, is the nearest settlement (<1 km) to the dam with a population of 3608 in 2012 (Sabure Health Center, unpublished report). Inhabitants live close to their irrigated fields. Meli (936 m asl), hereafter referred as lowland non-dam village, is located 15 km from the Kesem reservoir shoreline and was selected as a control village (beyond the vector impact range of the reservoir). Most of the inhabitants of both villages were agrarians, but only dam villages practiced irrigation. According to the local health center, malaria is a serious health problem in this region with intensive seasonal malaria transmission (Oromia Health Bureau, unpublished report).

Koka Dam (8°28′ N, 39°9′ E), hereafter referred as the midland dam, is located 100 km south-east of Addis Ababa, in the midland region of Central Ethiopia. The area is classified as semi-arid with 600 to 800 mm of annual rainfall, a mean annual temperature of 20 °C, and unstable/seasonal malaria transmission (National Meteorology Agency, unpublished report). Commissioned in 1969, Koka Dam was constructed to provide 46 MW of hydro-electricity to Addis Ababa and has a capacity of 1850 million m^3^. Ejersa (1564 m asl; population 4236), hereafter referred to as the midland dam village, is a rural village located adjacent (<1 km) to the Koka Reservoir shoreline. No irrigation was practiced around Koka. Dubsi (1566 m asl; population 3,421; Adama Health Center, unpublished report), hereafter referred as the midland non-dam village, is located about 10 km from Koka Reservoir, and was used as a control village. The inhabitants of both villages are largely agrarians. Malaria is common in both villages, with the main transmission season occurring from September to November, immediately following the long rainy season (June–August).

Koga Dam (11°25′ N, 37°09′ E), hereafter referred as the highland dam, is located on the Koga River, one of the major tributaries of the Blue Nile River in northwest Ethiopia, 556 km from Addis Ababa. The area is classified as a highland with an average annual rainfall of 1500 mm and has a mean annual temperature of 18 °C (National Meteorological Agency, unpublished report). Koga Dam has a storage capacity of 83.1 million m^3^ with the reservoir inundating an area of 17.5 km^2^. Commissioned in 2009, Koga Dam was constructed to provide water security for 7000 ha of irrigated land growing wheat, corn, and teff downstream of the dam. Endemir (1955 m asl), hereafter referred as the highland dam village, is located adjacent (<1 km) to the reservoir shoreline and had a population of 2907 in 2013 (Merawi Health Center, unpublished report). Sira Behibret (1942 m asl), hereafter referred as the highland non-dam village, is located 12 km from the reservoir shoreline and had a population of 3241 in 2013 (Merawi Health Center, unpublished report). The inhabitants of both villages are agrarians, and cattle herding is also common. Only dam villages use irrigation.

Vector control that involves use of bed nets and indoor residual spraying is commonly practiced in all study villages. No socioeconomic or intervention differences was observed among study villages. *Plasmodium falciparum* is the most common malaria parasite species, causing between 60 and 80% of malaria cases in the study area, with *Plasmodium vivax* causing the remaining malaria illnesses [12]. *Anopheles arabiensis* (the most widely distributed *An. gambiae sensu lato* species in Ethiopia) is the major malaria vector species, while *An. pharoensis* plays a secondary role [16]. In this study, it was assumed that all *An. gambiae s.l.* collected were *An. arabiensis* based on previous PCR identifications [22, 23].

### Clinical malaria data collection

To assess the risk of malaria around dams, monthly data of retrospective microscope-confirmed malaria cases were obtained from health centers in each of the three dam sites (2010–2014). Inhabitants of the study area commonly visit these government health facilities for medical consultation since they provide medical services free of charge. The malaria dataset was sorted for each of the six villages (three dam and three non-dam villages) and species of malaria parasite as confirmed by microscopy.

### Mosquito sampling

Larval and adult mosquitoes were sampled every 3 weeks in each of the study villages from October 2013 to July 2014. Larval stages were sampled from any water body such as rain pools, man-made pools, reservoir shoreline puddles, irrigation canals, and irrigated field paddies. During each survey, all potential mosquito breeding habitats within a 1-km radius of the study village were sampled using 350 mL standard dippers [24]. First, the surface area of the each potential mosquito breeding site was estimated in square meters (m^2^), and sampling was undertaken at a rate of six dips per m^2^ [24]. At least one dip was taken when the breeding habitat was too small (<0.2 m^2^). Larval anopheline samples were then counted and stored in vials by direct pipetting, killed by gentle heating, and preserved in 70% alcohol for later taxonomic identification. Larval samples from each mosquito habitat were placed in separate vials. Preserved larval anophelines were identified to species by microscope in the laboratory using morphological characteristics [25].

Adult mosquitoes were collected using CDC light traps (Model 512; J W Hock Co, Atlanta, USA). In each study village, a total of ten light traps were deployed for overnight mosquito collection from 1800 to 0630 h. Five of the light traps were deployed inside human homesteads and the other five were installed outdoors. Houses for light trap mosquito collection were randomly selected, and sampling was conducted in the same houses throughout the period of the study. Each indoor light trap was placed in a bedroom, near a wall, with the bulb about 50 cm above a person sleeping under an untreated bed net [15]. The outdoor light traps were installed on trees nearby open cattle enclosures where some of the villagers spent the evening. The following morning, light traps were collected and emptied into paper boxes containing a silica gel desiccant. Anophelines were later sorted out from other mosquito taxa, counted, and identified to species in the laboratory using morphological characteristics [26]. Female anophelines were kept at room temperature (22–25 °C) with silica gel desiccator until processed.

### Mosquito processing

The head-thorax portion of each dried female anopheline was processed to detect *P. falciparum* circumsporozoite antigens using enzyme-linked imminosorbent assay (ELISA) [27]. To determine mosquito blood meal sources (human vs bovine), the abdomen portion of blood-engorged female anophelines was tested using the direct ELISA technique [28].

### Statistical analysis

Monthly malaria incidence was express as the number of microscope-confirmed malaria cases in a given month per 1000 population [29]. Larval and adult counts were log-transformed before analysis to normalize the data. Anopheline larval density was determined as the mean number of anopheline larvae per square meter. Adult mosquito density was expressed as the mean number of adult mosquitoes per light trap per night, separated for indoor and outdoor traps within each study village. Differences in malaria incidence and larval and adult mosquito densities were tested among the elevation settings (pairing reservoir and non-reservoir villages at each site) using one-way analysis of variance (ANOVA) with post hoc HSD Tukey’s test applied to test differences between villages within each dam site [30].

The sporozoite rate was expressed as the proportion of mosquitoes positive for *Plasmodium* sporozoites from the total number of mosquitoes of a species tested by ELISA. Human biting rates were derived from light trap catches by dividing adult anopheline density by a factor of 1.5 to match light trap catches with human landing catches, as determined by Yohannes et al. [15]. The human biting rate was then multiplied by the sporozoite rate to estimate the entomological inoculation rate (EIR). For each *Anopheles* species, the human blood index (HBI) was determined as the proportion of samples positive for human blood from the total samples tested by blood meal ELISA. All analyses were done using Microsoft Excel 2010 and SPSS statistical software version 22 (SPSS Inc, Chicago, IL, USA). The level of significance used for all tests was 0.05.

## Results

### Malaria incidence

**Fig. 2:**
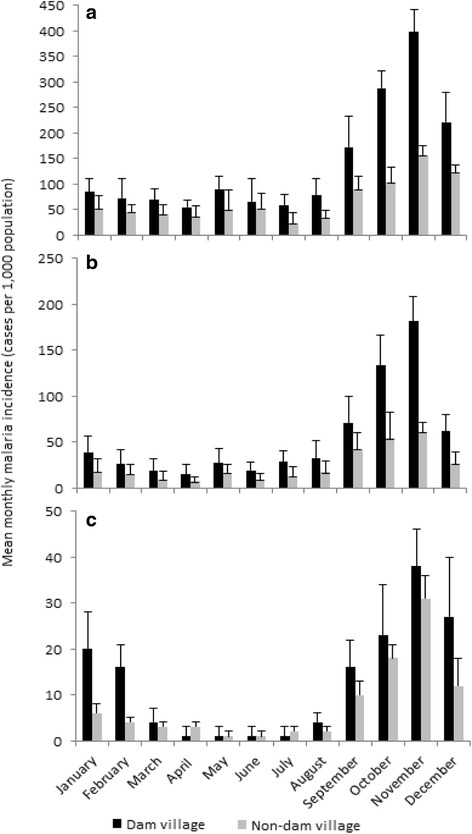
Mean monthly malaria incidence (cases per 1000 population) in the **a** lowland, **b** midland, and **c** highland dam and non-dam villages in Ethiopia, 2010–2014. Note the different scales on the *Y*-axes. Error bars are the standard error

The mean monthly malaria incidence was generally higher in the dam villages than in the non-dam villages in all three study dam sites (degree of freedom (df) = 2; *P* < 0.05) (Fig. 2). The mean monthly malaria incidence at the lowland dam village (mean = 137.4; 95% CI = 86.3–188.5) was twofold higher than at the midland dam village (54.4; 95% CI = 38.2–70.6) and over tenfold higher than at the highland dam village (12.7; 95% CI = 10.2–15.2). A significant difference in malaria incidence among the three dam sites was found (ANOVA df = 2; *F* = 5.673; *P* < 0.05). The peak period of malaria incidence at all study sites was between September and November.

### Mosquito breeding sites and larval density

A total of 1838 potential mosquito larval sites were surveyed during the study period (Table 1). Of these, 1556 (84.7%) were encountered in the dam villages, while only 282 (15.3%) were from non-dam villages. Anopheline larvae were detected at 454 (29.2%) and 43 (15.2%) of these sites at dam and non-dam villages, respectively. At the lowland, the number of positive anopheline breeding sites was over 12 times higher in the dam village (*n* = 271) than in the non-dam village (*n* = 22). At the midland, the number of positive anopheline breeding sites was nearly nine times higher in the dam village (*n* = 115) than in the non-dam village (*n* = 16). Similarly, the number of anopheline breeding sites was six times higher in the dam village (*n* = 68) than in the non-dam village (*n* = 11).

**Table 1:** Summary of anopheline larval surveys conducted in the lowland (Kesem), midland (Koka) and highland (Koga) dam and non-dam villages in Ethiopia between October 2013 and July 2014

	Village	No. of potential anopheline breeding sites	No. of positive anopheline breeding sites	Total area of potential mosquito breeding sites (m^2^)	Total area of positive anopheline breeding sites (m^2^)	Total no. of anopheline larvae sampled	Mean larval density (no. of larvae per m^2^)
Lowland dam	Dam village	712	271	202.1	153.4	793	10.8
	Non-dam village	148	22	71.6	56.3	398	3.7
Midland dam	Dam village	508	115	164.2	116.5	308	5.1
	Non-dam village	83	16	64.1	48.1	122	1.4
Highland dam	Dam village	336	68	121.5	84.2	165	0.5
	Non-dam village	51	11	39.8	18.5	74	0.2

A total of 354.1 and 122.7 m^2^ of water body was found supporting anopheline mosquito breeding in the dam and non-dam villages, respectively. The area of anopheline breeding sites was 1.3 and 1.8 times higher in the lowland dam village compared to that in the midland and highland dam villages, respectively. The surface area of anopheline larval sites in the dam villages was generally 3–5 times higher than in the non-dam villages.

A total of 1860 anopheline larvae were sampled during the period of the study (Table 1). Of which, the majority (64%; *n* = 1,191) were sampled from the lowland dam while the rest 23% (*n* = 430) and 13% (*n* = 239) were from the midland and highland dam sites, respectively. Anopheline larval abundance was generally higher in the dam villages than in the non-dam villages at all study dam sites. The mean larval density (larvae per square meter) was significantly higher in the lowland dam village (ANOVA mean = 10.8; 95% CI = 7.9–13.7; *F* = 31.413; *P* < 0.01) than in the midland (mean = 5.1; 95% CI = 4.0–6.2) and highland (mean = 0.5; 95% CI = 0.3–0.7) dam villages. Overall, controlling for elevation differences, the variation in mean larval density among the three dam sites was significant (ANOVA *F* = 8.453; *P* < 0.01).

Five *Anopheles* species were identified as larvae across the study area: *An. gambiae s.l.* (hereafter referred as *An. arabiensis)*, *An. pharoensis*, *An. funestus s.l.*, *An. coustani s.l.*, and *An. cinereus* (Table 2). Among these, larvae of *An. arabiensis* was predominant in all study villages, accounting for 58% (*n* = 1083) of total larval collections, followed by *An. pharoensis* (24%; *n* = 453), *An. coustani s.l.* (10%; *n* = 189), and *An. funestus s.l.* (7%; *n* = 131). Larvae of *An. funestus s.l.* were found only in the lowland dam village, predominantly in the shoreline puddles and irrigation canals. In the lowland setting, *An. arabiensis* was predominantly found in irrigation canals and shoreline puddles, contributing to 56 and 37% of the total larval collection from these habitats, respectively. Shoreline puddles accounted for 70.1% of this species’ larvae in the midland dam village, while irrigation canals and shoreline puddles accounted for 49.5 and 12.6% in the highland dam village, respectively. Similarly, *An. pharoensis* larvae were primarily found within irrigation canals and/or shoreline puddles at each of the dam villages. In control villages, larval *An. arebiensis* was predominant, commonly found in rain pools and man-made pools. Overall, while anopheline larvae at the midland dam village were collected mainly from shoreline puddles, both shoreline puddles and irrigation canals were the dominant larval habitats at the lowland and highland dam villages.

**Table 2:** Distribution of *Anopheles* species across different types of larval breeding habitats in the lowland (Kesem), midland (Koka), and highland (Koga) dam and non-dam villages in Ethiopia, between October 2013 and July 2014

Site	Village	Type of breeding habitat	Number of positive mosquito breeding sites	Area of positive mosquito breeding sites sampled (m^2^)	*An. arabiensis*	*An. pharoensis*	*An. coustani*	*An. funestus*	*An. cinereus*	Total no. of *Anopheles* larvae found
Lowland dam	Dam village	Shoreline puddle	188	77.3	164	75	10	61	0	310
		Rain pools	12	15.2	26	6	5	0	2	39
		Man-made pools	8	4.9	8	0	9	0	1	18
		Irrigation canals	63	56	248	89	26	63	0	426
		Total	271	153.4	446	170	50	124	3	793
	Non-dam village	Shoreline puddle	-^a^	-	-	-	-	-	-	-
		Rain pools	18	49	177	110	51	0	0	338
		Man-made pools	4	7.3	31	17	12	0	0	60
		Irrigation canals	-	-	-	-	-	-	-	-
		Total	22	56.3	208	127	63	0	0	398
Midland dam	Dam village	Shoreline puddle	90	84.8	129	84	19	4	0	236
		Rain pools	10	17.2	18	2	3	0	0	23
		Man-made pools	15	14.5	37	3	8	0	1	49
		Irrigation canals	-	-	-	-	-	-	-	-
		Total	115	116.5	184	89	30	4	1	308
	Non-dam village	Shoreline puddle	-	-	-	-	-	-	-	-
		Rain pools	12	37.1	47	27	9	0	0	83
		Man-made pools	4	11	32	5	2	0	0	39
		Irrigation canals	-	-	-	-	-	-	-	-
		Total	16	48.1	79	32	11	0	0	122
Highland dam	Dam village	Shoreline puddle	10	12.1	14	2	6	1	0	23
		Rain pools	17	15.8	28	0	10	0	0	38
		Man-made pools	2	1.5	4	0	2	0	0	6
		Irrigation canals	28	54.8	65	17	14	2	0	98
		Total	57	84.2	111	19	32	3	0	165
	Non-dam village	Shoreline puddle	-	-	-	-	-	-	-	-
		Rain pools	8	12.4	47	11	3	0	0	61
		Man-made pools	3	6.1	8	5	0	0	0	13
		Irrigation canals	0	0	0	0	0	0	0	0
		Total	11	18.5	55	16	3	0	0	74

^a^- indicates that this type of breeding habitat did not exist

**Fig. 3:**
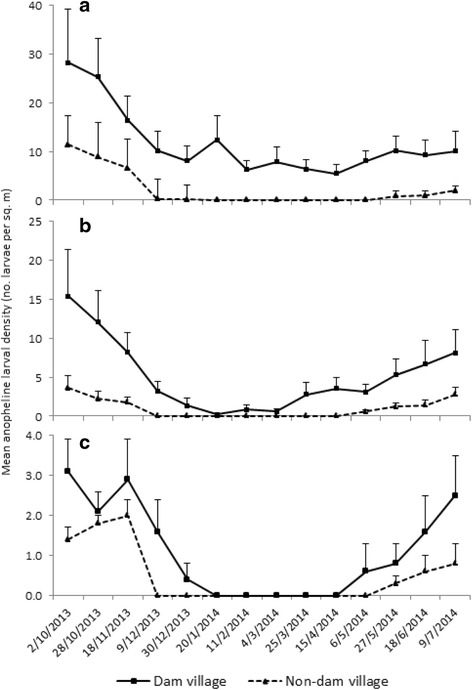
Mean anopheline larval density (no. larvae per m^2^) in the **a** lowland, **b** midland, and **c** highland dam and non-dam villages in Ethiopia, between October 2013 and July 2014. Note the different scales on the *Y*-axes. Error bars are the standard error

Anopheline larval density peaked between October and November, dropping during the dry season, and building up to the wet season in all study villages (Fig. 3). However, overall mean larval density was generally higher in the dam villages than in the non-dam villages at all three study areas.

### Adult mosquito abundance

A total of 5140 adult anopheline mosquitoes were collected during the study period. Of these, 68% (*n* = 3503), 29% (*n* = 1506), and 3% (*n* = 131) were from lowland, midland, and highland study sites, respectively (Table 3). *Anopheles arabiensis* was the predominant species in all villages, accounting for 53% of the total adult anopheline collections. *Anopheles pharoensis* was the next most abundant species (31%), followed by *An. funestus s.l.* (9.4%), *An. coustani s.l.* (6.4%), and *An. cinereus* (0.2%).

**Table 3:** Number and mean density of adult anophelines and collected in the lowland (Kesem), midland (Koka), and highland (Koga) dam and non-dam villages in Ethiopia, between October 2013 and July 2014

		*An. arabiensis*		*An. pharoensis*		*An. coustani*		*An. funestus*		*An. cinereus*		Total Anophelines	
		No. (%)	Mean density^a^	No. (%)	Mean density	No. (%)	Mean density	No. (%)	Mean density	No. (%)	Mean density	No. (%)	Mean density
Lowland dam	Dam village	1423	15.81	782	8.69	99	1.10	449	4.99	2	0.02	2755	30.61
	Non-dam village	466	5.18	251	2.79	31	0.34	0	0.00	0	0.00	748	8.31
Midland dam	Dam village	541	6.01	421	4.68	103	1.14	36	0.40	8	0.09	1109	12.32
	Non-dam village	205	2.28	126	1.40	66	0.73	0	0.00	0	0.00	397	4.41
Highland dam	Dam village	64	0.71	16	0.18	12	0.13	0	0.00	0	0.00	92	1.02
	Non-dam village	23	0.26	0	0.00	16	0.18	0	0.00	0	0.00	39	0.43
Total	Dam village	2028	7.51	1219	4.51	214	0.79	485	1.80	10	0.04	3.956	14.65
	Non-dam village	694	2.57	377	1.40	113	0.42	–	–	0	0.00	1.184	4.39

^a^Mean density refers to the mean number of adult anophelines per trap per night during the sampling period

**Fig. 4:**
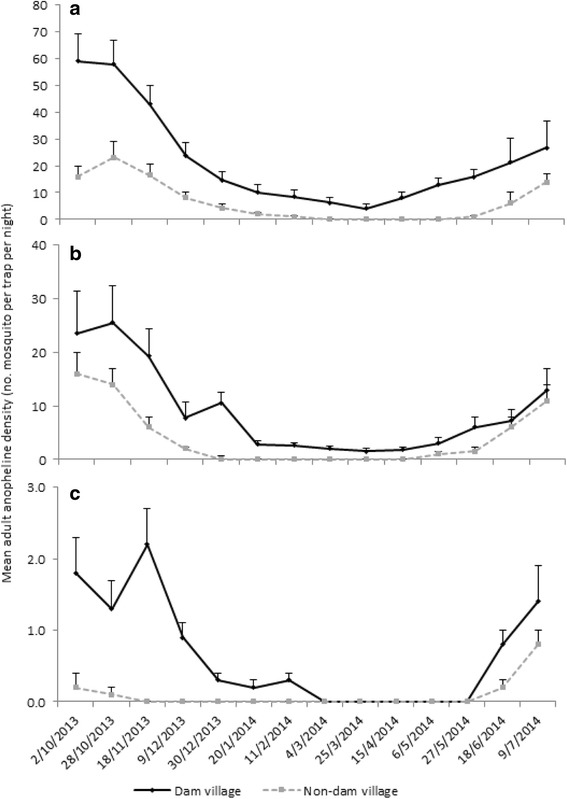
Mean adult anopheline density (number of mosquitoes per trap per night) in the **a** lowland, **b** midland, and **c** highland dam villages and non-dam villages in Ethiopia, between October 2013 and July 2014. Note the different scales on the *Y*-axes. Error bars are the standard error

Similar to larvae, anopheline adult density peaked between October and November, fell during the dry season, and increased again in June with the commencement of the wet season in all study villages (Fig. 4). Overall mean adult anopheline density varied significantly across villages (ANOVA *F* = 23.89; *P* < 0.001) and was generally higher at the dam villages than at the non-dam villages in all three study areas. The highest density was recorded at the lowland dam village (mean = 10.8 anopheline per trap per night; 95% CI = 6.2–15.4) and the lowest at the highland non-dam village (mean = 0.2; 95% CI = 0.1 – 0.4). Similarly, the overall mean adult anopheline density at the lowland dam village was 2.2 times higher than at the midland dam village and 22 times higher than at the highland dam village.

Indoor and outdoor adult mosquito sampling detected *An. arabiensis* predominantly indoors in all study villages (Table 4). The density of *An. pharoensis* was also higher indoors than outdoors at the lowland dam site but not at the midland and highland dam sites. In contrast, *An. coustani s.l.* and *An. funestus s.l.* were predominantly sampled from outdoor traps in all study sites.

**Table 4:** Indoor and outdoor mean adult anopheline density (no. of mosquitoes per trap per night) in the lowland (Kesem), midland (Koka), and highland (Koga) dam and non-dam villages in Ethiopia, between October 2013 and July 2014

Study site		*An. arabiensis*		*An. pharoensis*		*An. coustani*		*An. funestus*		*An. cinereus*		Total	
		Indoor	Outdoor	Indoor	Outdoor	Indoor	Outdoor	Indoor	Outdoor	Indoor	Outdoor	Indoor	Outdoor
Lowland	Dam village	20.87	10.76	10.13	7.24	0.71	1.49	0.91	9.07	0.00	0.04	32.62	28.60
	Non-dam village	6.89	3.47	4.18	1.40	0.22	0.47	0.00	0.00	0.00	0.00	11.29	5.33
Midland	Dam village	7.20	4.82	2.51	6.84	0.47	1.82	0.16	0.64	0.00	0.18	10.33	14.31
	Non-dam village	3.11	1.44	1.04	1.76	0.53	0.93	0.00	0.00	0.00	0.00	4.69	4.13
Highland	Dam village	1.13	0.29	0.13	0.22	0.07	0.20	0.00	0.00	0.00	0.00	1.33	0.71
	Non-dam village	0.31	0.20	0.00	0.00	0.09	0.27	0.00	0.00	0.00	0.00	0.40	0.47
Total	Dam village	29.20	15.87	12.78	14.31	1.24	3.51	1.07	9.71	0.00	0.22	44.29	43.62
	Non-dam village	10.31	5.11	5.22	3.16	0.84	1.67	0.00	0.00	0.00	0.00	16.38	9.93

### Blood meal sources and entomological inoculation rate

ELISA results indicated that *An. funestus s.l.* (human blood index (HBI) = 87.2%) and *An. arabiensis* (HBI = 82.4%) were the most anthropophagic species in the lowland dam village (Table 5). Slightly lower HBI values (70.7–72.7%) were recorded for *An. arabiensis* in the other dam villages. In contrast, the proportion of blood meals of *An. arabiensis* originating from bovine sources appeared to increase from lowland (22%) to midland (34%) and highland (36%). *An. pharoensis* preferred human blood meals over bovine sources, while *An. coustani s.l.* preferred bovine over human blood in all study villages.

**Table 5:** Blood meal sources of female anophelines in the lowland (Kesem), midland (Koka), and highland (Koga) dam and non-dam villages in Ethiopia, between October 2013 and July 2014

Site	Village	*An. arabiensis*	*An. pharoensis*	*An. funestus*	*An. coustani*
Lowland dam	Dam village				
	No. tested	924	508	311	47
	Positive for human blood (%)	761 (82.4)	348 (68.5)	272 (87.5)	18 (38.3)
	Positive for bovine blood (%)	203 (22.0)	209 (41.1)	40 (12.9)	32 (68.1)
	Unidentified (%)	9 (1.0)	5 (1.0)	2 (0.6)	3 (6.4)
	Non-dam village				
	No. tested	278	199	0	20
	Positive for human blood (%)	202 (72.7)	122 (61.3)	0	6 (30.0)
	Positive for bovine blood (%)	88 (31.7)	72 (36.2)	0	15 (75.0)
	Unidentified (%)	4 (1.4)	7 (3.5)	0	1 (5.0)
Midland dam	Dam village				
	No. tested	392	314	18	73
	Positive for human blood (%)	277 (70.7)	201 (64.0)	11 (61.1)	28 (38.4)
	Positive for bovine blood (%)	135 (34.4)	123 (39.2)	7 (38.9)	54 (74.0)
	Unidentified (%)	14 (3.6)	17 (5.4)	0 (0)	3 (4.1)
	Non-dam village				
	No. tested	168	91	0	44
	Positive for human blood (%)	118 (70.2)	61 (67.0)	0	18 (40.9)
	Positive for bovine blood (%)	62 (36.9)	32 (35.2)	0	34 (77.3)
	Unidentified (%)	7 (4.2)	3 (3.3)	0	2 (4.5)
Highland dam	Dam village				
	No. tested	45	9	0	5
	Positive for human blood (%)	32 (71.1)	6 (66.7)	0	2 (40.0)
	Positive for bovine blood (%)	16 (35.6)	4 (44.4)	0	3 (60.0)
	Unidentified (%)	0 (0.0)	1 (11.1)	0	0 (0.0)
	Non-dam village				
	No. tested	11	0	0	16
	Positive for human blood (%)	8 (72.7)	0	0	7 (43.8)
	Positive for bovine blood (%)	4 (36.4)	0	0	9 (56.3)
	Unidentified (%)	1 (9.1)	0	0	1 (6.3)

A total of 4848 female anophelines were tested for *P. falciparum* sporozoite infections (Table 6). The highest sporozoite infection rate was detected in the lowland dam village where 4.5% (20/449), 4.1% (59/1423), and 2.3% (18/782) of *An. funestus s.l.*, *An. arabiensis*, and *An. pharoensis*, respectively, were found to be positive. In the lowland non-dam village, 1.7% (8/466) of *An. arabiensis* and 1.2% (3/251) of *An. pharoensis* tested positive for *P. falciparum* sporozoites. In the midland dam village, the sporozoite rate of *An. arabiensis* and *An. pharoensis* was 2% (11/541) and 1.4% (6/421), respectively, while only a single female *An. arabiensis* (0.5%, 1/205) tested positive for *P. falciparum* sporozoite in the midland non-dam village. All sporozoite-infected anophelines were collected during the main transmission season. None of the samples from the highland dam villages tested positive for *P. falciparum* sporozoites.

**Table 6:** *Plasmodium falciparum* sporozoite rate and annual entomological inoculation rate (EIR) of *Anopheles* mosquitoes in the lowland (Kesem), midland (Koka), and highland (Koga) dam and non-dam villages in Ethiopia, between October 2013 and July 2014

Site	Village	*An. arabiensis*	*An. pharoensis*	*An. funestus*	*An. coustani*
Lowland dam	Dam village				
	No. tested	1423	782	449	99
	No. positive (%)	59 (4.1)	18 (2.3)	20 (4.5)	0 (0.0)
	Annual EIR	129.8	33.3	47.8	0
	Non-dam village				
	No. tested	466	251	36	103
	No. positive (%)	8 (1.7)	3 (1.2)	0 (0.0)	0 (0.0)
	Annual EIR	15.6	5.0	0	0
Midland dam	Dam village				
	No. tested	541	421	36	103
	No. positive (%)	11 (2.0)	6 (1.4)	0 (0.0)	0 (0.0)
	Annual EIR	20.7	10.2	0	0
	Non-dam village				
	No. tested	205	126	−^a^	66
	No. positive (%)	1 (0.5)	0 (0.0)	−	0 (0.0)
	Annual EIR	2.0	0	−	0
Highland dam	Dam village				
	No. tested	64	16	−	12
	No. positive (%)	0 (0.0)	0 (0.0)	−	0 (0.0)
	Annual EIR	0	0		0
	Non-dam village				
	No. tested	23	0	0	16
	No. positive	0 (0.0)	−	−	0 (0.0)
	Annual EIR	0	0	0	0

EIR refers to the number of infective bites per person per year

^a^− (minus) refers to absence of the species in that area

The annual entomological inoculation rate (EIR) for *An. arabiensis*, *An. funestus s.l.*, and *An. pharoensis* at the lowland dam village was found to be 129.8, 47.8, and 33.3 infective bites per person per year (ib/p/y), respectively (Table 6). In contrast, the annual EIR of *An. arabiensis* and *An. pharoensis* at the lowland non-dam village was 15.6 and 5.0 ib/p/y, respectively. At the midland dam village, the annual EIR was found to be 20.7 and 10.2 ib/p/y by *An. arabiensis* and *An. pharoensis*, respectively, while the annual EIR in the midland non-dam village was 2.0 ib/p/y by *An. arabiensis*. Overall, the data revealed that dams resulted in 10 and 15-fold increases in EIR in the lowland and midland areas, respectively, compared to that in the non-dam villages in the same settings.

## Discussion

The present study indicates the link between dams and malaria in different eco-epidemiological settings in Ethiopia. At the lowland and midland settings, reservoir shoreline puddles and irrigation canals were the major malaria vector breeding habitats, contributing to 70–80% of the anopheline larval breeding sites. *Anopheles arabiensis* and *An. pharoensis* were the major malaria vectors, occurring in higher abundance at lowland and midland dam villages than at the highland dam villages.

*Anopheles arabiensis* and *An. pharoensis* were primarily breeding in shoreline puddles and irrigation canals in the lowland, midland, and highland dam villages, although their abundance differed among villages. The abundance of these vector species peaked between October and November when the water level started receding following peak water level between July and August: this creates breeding sites at the shorelines. A previous study around the Koka Dam indicated that while *An. arabiensis* prefers shallow sunlit shoreline puddles, *An. pharoensis* breeds in semi-permanent and partly covered large water pools [16]. A preference for similar breeding habitats was documented for these species in the neighboring Ziway area [31], around microdams in northern Ethiopia [15] and elsewhere in Ethiopia [11, 32].

Larval and adult vector densities decreased from the lowland to midland to highland dam villages. Climate variables such as temperature are the major factors that determine rates of mosquito breeding, adult longevity, and malaria parasite development at different elevation settings [33]. Dams in lowland areas create ideal breeding sites for mosquitoes where rainfall is the limiting factor underpinning the availability of mosquito breeding habitats. Moreover, irrigation activities increase vector breeding habitats by creating waterlogged sites in the irrigated fields as well as irrigation canals. A previous study in central Ethiopia where irrigation is commonly practiced indicated that poor irrigation water management led to increased mosquito breeding habitats with a high risk of malaria transmission [34]. A number of studies in sub-Saharan Africa have also revealed the link between irrigation and malaria and the need for targeted planning and implementation of mosquito control measures in order to reduce mosquito breeding [35–40]. Similarly, the present study identified that the lowland irrigation dam had increased mosquito vector abundance and malaria transmission.

A higher vector density along with a high HBI and EIR in the lowland dam village revealed the serious potential negative impact of dams on malaria in lowland Ethiopia. Moreover, the presence of three vector species (*An. arabiensis*, *An. pharoensis*, and *An. funestus s.l.*) with a combined annual EIR of 210 per person per year around the Kesem Dam highlights the pressing need to devise vector control strategies around lowland dams. Nevertheless, the annual EIR of *An. arabiensis* (129.8) and *An. funestus s.l.* (47.4) in the lowland dam village was lower than those reported (314 and 88, respectively) from the Lower Moshi irrigation area of northern Tanzania for the same species [41]. The annual EIR in the midland dam village was comparable to previously documented EIR in the same study area [16] and in western [32] and southwestern Ethiopia [42]. The higher EIR and malaria incidence in the lowland dam village is likely to have been driven by the greater climatic suitability of lowland areas for malaria transmission. Moreover, due to high humidity in the lowland dam area, people often sleep outside under trees (personal observation), which increases the chance of mosquito bites as bed nets are not suitable for outdoor use. Additional malaria intervention measures are thus required particularly for outdoor-dominant *An. funestus s.l.* in the lowland dam village and *An. pharoensis* in the midland dam village since the current malaria intervention strategies entirely target indoor mosquitoes.

The present study has documented for the first time in four decades the role of *An. funestus s.l.* in malaria transmission in the lowland regions of Ethiopia. This species disappeared from several wetland areas of Ethiopia in the 1970s due to land use changes [32]. The presence of *P. falciparum* sporozoite-infected *An. funestus s.l.* along with a high HBI confirms the role of this species in malaria transmission in the lowland dam area. The reported high sporozoite rate of *An. funestus s.l.* (4.5%) in the lowland dam village was comparable to that reported from Uganda (5.3%) [43] but lower than that documented in Eritrea (9.5%) [44]. Krafsur [45] reported a lower sporozoite rate (1.1%) for this species in the wetlands of the Gambella Region in western Ethiopia. Erlanger et al. [46] reported that the density of *An. funestus s.l.* was 25-fold higher in the irrigated sites as compared to that in non-irrigated sites in the sub-Saharan Africa. The present study documented the link between *An. funestus s.l.* and irrigation dams in the lowland areas of Ethiopia which otherwise would not have been identified.

Malaria incidence and vector density peaked in all study villages between October and November immediately after the main wet season. The impact of the dams was to intensify the malaria transmission instead of extending the period of transmission. Similar findings were documented in the Gilgel-gibe Dam in south Ethiopia [13], the Bemendjin Dam of Cameroon [9], the Kamburu Dam of Kenya [10], the Mthera Dam of Tanzania [47], and the Usuma Reservoir of Nigeria [48]. A recent review work reported that dams intensify malaria transmission in seasonally unstable areas since they provide breeding habitats for mosquito vectors [5]. Reservoir shoreline puddles and irrigation canals provide suitable breeding habitats for malaria vector mosquitoes around dam sites. A previous study around Koka Dam indicated that lower water level drawdown rates between September and November led to increased formation of reservoir shoreline puddles [12]. Reservoir water management is thus crucial to minimize the presence and persistence of reservoir shoreline puddles. In addition, irrigation canals do not operate during the wet season as farmers use rainfed agriculture during this period, leaving irrigation canals waterlogged from rainfall and thus providing ideal breeding grounds for malaria vector mosquitoes (personal observation). Draining irrigation canals and reducing water logging in agricultural fields were previously shown to be effective in reducing larval breeding habitats around microdams in northern Ethiopia [15]. Reservoirs need to be operated in such a way to suppress mosquito breeding as previously done in Tennessee Valley of the USA where faster drawdown of reservoir water levels were associated with lower larval mosquito abundance at the shoreline of the reservoir [49].

This study did not include data for all months of a year. For instance, September lies in the peak malaria transmission season and its inclusion would improve the seasonality of malaria transmission in each eco-epidemiological settings. Future longitudinal research is required to verify the results of this study and assess interannual water level variations on malaria transmission across dams in different ecological settings. Similarly, molecular work is needed to provide the taxonomic resolution for the species of the *An. funestus* complex in the lowland regions of Ethiopia.

## Conclusions

The link between dams and malaria must be considered while planning, designing, and operating large dams in sub-Saharan Africa where malaria is a primary public health challenge. The present study confirmed that dams in semi-arid lowland and midland areas intensify malaria transmission due to mosquito vector breeding in associated shoreline and irrigation habitats. However, such an effect was not detected at the highland dam area. Proper management of dams and associated shallow shoreline and canal habitats is thus essential to reduce malaria vector breeding around these economically important water infrastructures. Such environmental management techniques along with conventional vector interventions should be targeted to reduce malaria transmission around these critical infrastructures.

## Abbreviations

ANOVA: Analysis of variance
ASL: Above sea level
DF: Degree of freedom
EIR: Entomological inoculation rate
ELISA: Enzyme-linked immunosorbent assay
ib/p/y: Infective bites per person per year
